# Supplementary material for: Chemical Composition, Antioxidant and Antimicrobial Activity of Some Types of Honey from Banat Region, Romania
Source: Molecules. 2022 Jun 29;27(13):4179. doi: 10.3390/molecules27134179 (PMC9268046; doi:10.3390/molecules27134179)
Supplement: Supplementary file 1 [file molecules-27-04179-s001.zip › molecules-1774084-supplementary.pdf]

Table S1. MIC recorded for the ATCC strains tested

S. pyogenes (ATCC 19615)	*S. aureus* (ATCC 25923)	*S. flexneri* (ATCC 12022)	*P. aeruginosa* (ATCC 27853)	*E. coli* (ATCC 25922)	*S. typhimurium* (ATCC 14028)	*H. influenzae* (ATCC 10211)	*C. parapsilopsis* (ATCC 22019)	*C. albicans* (ATCC 10231)	*L. monocytogenes* (ATCC 19114)	*B. cereus* (ATCC 10876)
M1 10%	M1 10%	M1 10%	M1 10%	M1 10%	M1 10%	M1 10%	M1 10%	M1 10%	M1 10%	M1 10%
M1 15%	M1 15%	M1 15%	M1 15%	M1 15%	M1 15%	M1 15%	M1 15%	M1 15%	M1 15%	M1 15%
M1 20%	M1 20%	M1 20%	M1 20%	M1 20%	M1 20%	M1 20%	M1 20%	M1 20%	M1 20%	M1 20%
M1 25%	M1 25%	M1 25%	M1 25%	M1 25%	M1 25%	M1 25%	M1 25%	M1 25%	M1 25%	M1 25%
M2 10%	M2 10%	M2 10%	M2 10%	M2 10%	M2 10%	M2 10%	M2 10%	M2 10%	M2 10%	M2 10%
M2 15%	M2 15%	M2 15%	M2 15%	M2 15%	M2 15%	M2 15%	M2 15%	M2 15%	M2 15%	M2 15%
M2 20%	M2 20%	M2 20%	M2 20%	M2 20%	M2 20%	M2 20%	M2 20%	M2 20%	M2 20%	M2 20%
M2 25%	M2 25%	M2 25%	M2 25%	M2 25%	M2 25%	M2 25%	M2 25%	M2 25%	M2 25%	M2 25%
M3 10%	M3 10%	M3 10%	M3 10%	M3 10%	M3 10%	M3 10%	M3 10%	M3 10%	M3 10%	M3 10%
M3 15%	M3 15%	M3 15%	M3 15%	M3 15%	M3 15%	M3 15%	M3 15%	M3 15%	M3 15%	M3 15%
M3 20%	M3 20%	M3 20%	M3 20%	M3 20%	M3 20%	M3 20%	M3 20%	M3 20%	M3 20%	M3 20%
M3 25%	M3 25%	M3 25%	M3 25%	M3 25%	M3 25%	M3 25%	M3 25%	M3 25%	M3 25%	M3 25%
M4 10%	M4 10%	M4 10%	M4 10%	M4 10%	M4 10%	M4 10%	M4 10%	M4 10%	M4 10%	M4 10%
M4 15%	M4 15%	M4 15%	M4 15%	M4 15%	M4 15%	M4 15%	M4 15%	M4 15%	M4 15%	M4 15%
M4 20%	M4 20%	M4 20%	M4 20%	M4 20%	M4 20%	M4 20%	M4 20%	M4 20%	M4 20%	M4 20%
M4 25%	M4 25%	M4 25%	M4 25%	M4 25%	M4 25%	M4 25%	M4 25%	M4 25%	M4 25%	M4 25%
M5 10%	M5 10%	M5 10%	M5 10%	M5 10%	M5 10%	M5 10%	M5 10%	M5 10%	M5 10%	M5 10%
M5 15%	M5 15%	M5 15%	M5 15%	M5 15%	M5 15%	M5 15%	M5 15%	M5 15%	M5 15%	M5 15%
M5 20%	M5 20%	M5 20%	M5 20%	M5 20%	M5 20%	M5 20%	M5 20%	M5 20%	M5 20%	M5 20%
M5 25%	M5 25%	M5 25%	M5 25%	M5 25%	M5 25%	M5 25%	M5 25%	M5 25%	M5 25%	M5 25%
M6 10%	M6 10%	M6 10%	M6 10%	M6 10%	M6 10%	M6 10%	M6 10%	M6 10%	M6 10%	M6 10%
M6 15%	M6 15%	M6 15%	M6 15%	M6 15%	M6 15%	M6 15%	M6 15%	M6 15%	M6 15%	M6 15%
M6 20%	M6 20%	M6 20%	M6 20%	M6 20%	M6 20%	M6 20%	M6 20%	M6 20%	M6 20%	M6 20%

M6 25%	M6 25%	M6 25%	M6 25%	M6 25%	M6 25%	M6 25%	M6 25%	M6 25%	M6 25%	M6 25%
M7 10%	M7 10%	M7 10%	M7 10%	M7 10%	M7 10%	M7 10%	M7 10%	M7 10%	M7 10%	M7 10%
M7 15%	M7 15%	M7 15%	M7 15%	M7 15%	M7 15%	M7 15%	M7 15%	M7 15%	M7 15%	M7 15%
M7 20%	M7 20%	M7 20%	M7 20%	M7 20%	M7 20%	M7 20%	M7 20%	M7 20%	M7 20%	M7 20%
M7 25%	M7 25%	M7 25%	M7 25%	M7 25%	M7 25%	M7 25%	M7 25%	M7 25%	M7 25%	M7 25%
M8 10%	M8 10%	M8 10%	M8 10%	M8 10%	M8 10%	M8 10%	M8 10%	M8 10%	M8 10%	M8 10%
M8 15%	M8 15%	M8 15%	M8 15%	M8 15%	M8 15%	M8 15%	M8 15%	M8 15%	M8 15%	M8 15%
M8 20%	M8 20%	M8 20%	M8 20%	M8 20%	M8 20%	M8 20%	M8 20%	M8 20%	M8 20%	M8 20%
M8 25%	M8 25%	M8 25%	M8 25%	M8 25%	M8 25%	M8 25%	M8 25%	M8 25%	M8 25%	M8 25%
M9 10%	M9 10%	M9 10%	M9 10%	M9 10%	M9 10%	M9 10%	M9 10%	M9 10%	M9 10%	M9 10%
M9 15%	M9 15%	M9 15%	M9 15%	M9 15%	M9 15%	M9 15%	M9 15%	M9 15%	M9 15%	M9 15%
M9 20%	M9 20%	M9 20%	M9 20%	M9 20%	M9 20%	M9 20%	M9 20%	M9 20%	M9 20%	M9 20%
M9 25%	M9 25%	M9 25%	M9 25%	M9 25%	M9 25%	M9 25%	M9 25%	M9 25%	M9 25%	M9 25%
M10 10%	M10 10%	M10 10%	M10 10%	M10 10%	M10 10%	M10 10%	M10 10%	M10 10%	M10 10%	M10 10%
M10 15%	M10 15%	M10 15%	M10 15%	M10 15%	M10 15%	M10 15%	M10 15%	M10 15%	M10 15%	M10 15%
M10 20%	M10 20%	M10 20%	M10 20%	M10 20%	M10 20%	M10 20%	M10 20%	M10 20%	M10 20%	M10 20%
M10 25%	M10 25%	M10 25%	M10 25%	M10 25%	M10 25%	M10 25%	M10 25%	M10 25%	M10 25%	M10 25%

[1] The red colour highlights the samples where the MIC was determined
